# Determinants of Scale-up From a Small Pilot to a National Electronic Immunization Registry in Vietnam: Qualitative Evaluation

**DOI:** 10.2196/19923

**Published:** 2020-09-22

**Authors:** Huyen Dang, Sang Dao, Emily Carnahan, Nami Kawakyu, Hong Duong, Trung Nguyen, Doan Nguyen, Linh Nguyen, Maya Rivera, Tuan Ngo, Laurie Werner, Nga Nguyen

**Affiliations:** 1 National Expanded Program on Immunization National Institute of Hygiene and Epidemiology Hanoi Vietnam; 2 PATH Hanoi Vietnam; 3 PATH Seattle, WA United States; 4 Department of Global Health University of Washington Seattle, WA United States

**Keywords:** immunization, immunization information system, electronic immunization registry, scale-up, digital health intervention, mHealth, eHealth

## Abstract

**Background:**

Digital health innovations can improve health system performance, yet previous experience has shown that many innovations do not advance beyond the pilot stage to achieve scale. Vietnam’s National Immunization Information System (NIIS) began as a series of digital health pilots, first initiated in 2010, and was officially launched nationwide in 2017. The NIIS is one of the few examples of an electronic immunization registry (EIR) at national scale in low- and middle-income countries.

**Objective:**

The aim of this study was to understand the determinants of scale-up of the national EIR in Vietnam.

**Methods:**

This qualitative study explored the facilitators and barriers to national scale-up of the EIR in Vietnam. Qualitative data were collected from October to December 2019 through in-depth key informant interviews and desk review. The mHealth Assessment and Planning for Scale (MAPS) Toolkit guided the development of the study design, interview guides, and analytic framework. MAPS defines the key determinants of success, or the “axes of scale,” to be groundwork, partnerships, financial health, technology and architecture, operations, and monitoring and evaluation.

**Results:**

The partnership and operations axes were critical to the successful scale-up of the EIR in Vietnam, while the groundwork and monitoring and the evaluation axes were considered to be strong contributors in the success of all the other axes. The partnership model leveraged complementary strengths of the technical working group partners: the Ministry of Health General Department of Preventive Medicine, the National Expanded Program on Immunization, Viettel (the mobile network operator), and PATH. The operational approach to introducing the NIIS with lean, iterative, and integrated training and supervision was also a key facilitator to successful scale-up. The financial health, technology and architecture, and operations axes were identified as barriers to successful deployment and scale-up. Key barriers to scale-up included insufficient estimates of operational costs, unanticipated volume of data storage and transmission, lack of a national ID to support interoperability, and operational challenges among end users. Overall, the multiple phases of EIR deployment and scale-up from 2010 to 2017 allowed for continuous learning and improvement that strengthened all the axes and contributed to successful scale-up.

**Conclusions:**

The results highlight the importance of the measured, iterative approach that was taken to gradually expand a series of small pilots to nationwide scale. The findings from this study can be used to inform other countries considering, introducing, or in the process of scaling an EIR or other digital health innovations.

## Introduction

Digital health innovations are changing the way health is delivered worldwide. The World Health Organization (WHO) defines digital health as “the field of knowledge and practice associated with the development and use of digital technologies to improve health” [1]. Digital health innovations can play an important role in improving health system performance and can advance progress toward achieving universal health coverage and sustainable development goals [2,3]. However, previous experience has shown that many digital health innovations do not advance beyond the pilot stage to become institutionalized within health systems [4-6].

Vietnam’s National Immunization Information System (NIIS) began as a series of digital health pilots, first initiated in 2010 [7], and was officially launched nationwide in 2017. By 2020, the NIIS included 20 million client records. The NIIS is an example of an electronic immunization registry (EIR), a confidential, computerized, population-based routine system to capture, store, access, and share individual-level, longitudinal health information on vaccine doses administered [8,9]. Immunization is among the most cost-effective child health interventions and saves 2 to 3 million lives per year; however, 1 in 5 infants do not receive all their required vaccine doses [10]. EIRs aim to improve the immunization delivery system to reach every child by supporting the delivery of more effective, efficient, data-driven care. EIRs can capture other individual-level demographic or health data and can link to other systems that manage vaccine stock and logistics, human resources, or other individual or population health data [11]. In Vietnam, the EIR, which includes SMS text message reminders, has been shown to improve immunization coverage and timeliness of vaccination [12] and is one of the few examples of an EIR at national scale in low- and middle-income countries.

Scale-up refers to “deliberate efforts to increase the impact of innovations successfully tested in pilot or experimental projects so as to benefit more people and to foster policy and program development on a lasting basis” [13]. Scaling goes beyond expanding an innovation to more users or geographies and in fact often results in new organizational or technological complexities [14]. Others have defined scale more expansively, including integration with the health system, sustainable funding and government support, and the ability to replicate, refine, and improve over time [4,15].

The digital health community has increasingly recognized the importance of scale and has initiated efforts to support scaling digital health interventions [15]. The Principles for Digital Development, collaboratively developed by 500+ implementers to capture best practices for integrating technology in development projects, highlight “design for scale” as one of 9 guiding principles [16]. Initiatives like the Health Data Collaborative [17], Digital Impact Alliance [18], and Digital Square [19] have been launched to support scale-up through alignment and coordination, strategic resourcing, operational guidance, and development of “global goods” that can be adapted and scaled in new contexts [15].

The evidence base to understand factors that influence scaling digital health interventions is limited [20,21]. The WHO and others have identified the need for implementation research to understand the complexities of implementing and scaling digital interventions [2,6]. National EIRs are far from being universal, even in high-income countries [22,23], and to our knowledge, there are limited studies of the facilitators and barriers influencing their scale-up, particularly in low-resource settings.

The aim of this study was to understand the determinants of scale-up of the national EIR in Vietnam, thereby contributing to the evidence on how and why digital interventions can successfully achieve scale.

## Methods

### Study Design

This study explored the facilitators and barriers to national scale-up of the EIR in Vietnam through qualitative methods, using the mHealth Assessment and Planning for Scale (MAPS) Toolkit as a conceptual framework [13]. The MAPS Toolkit, codeveloped in 2015 by the WHO, the United Nations Foundation, and Johns Hopkins University, outlines 6 axes to measure digital health project maturity. Qualitative data were collected through in-depth key informant interviews and document review and were analyzed according to the MAPS Toolkit axes.

### Conceptual Framework

**Figure 1:**
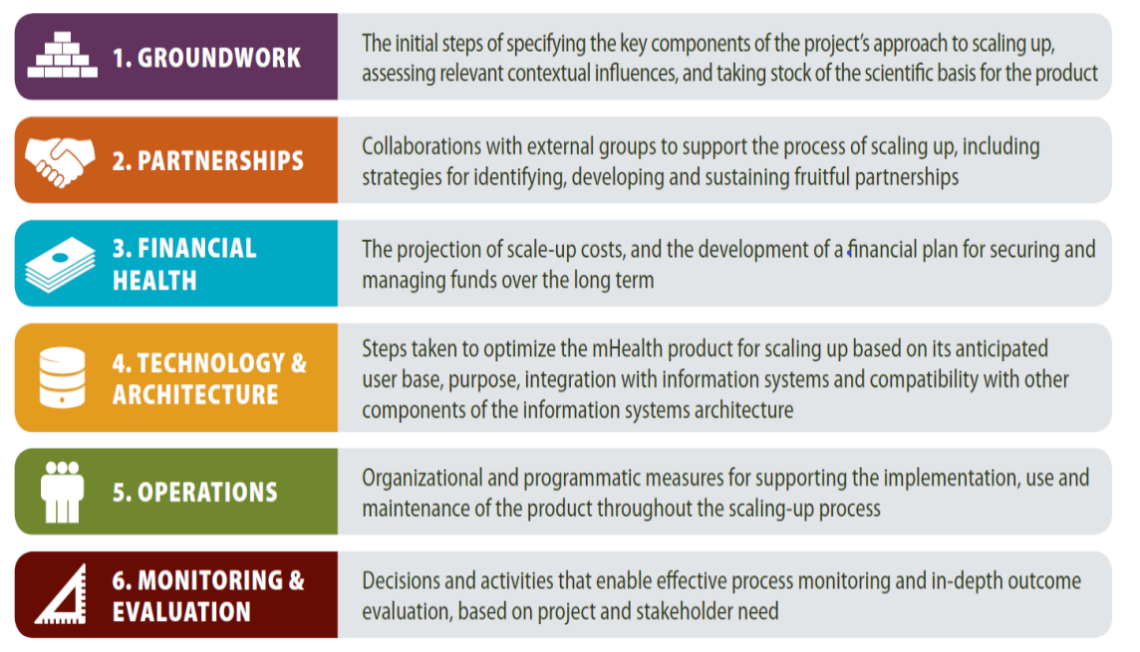
The mHealth Assessment and Planning for Scale Toolkit axes of scale [13]. mHealth: mobile health.

**Figure 2:**
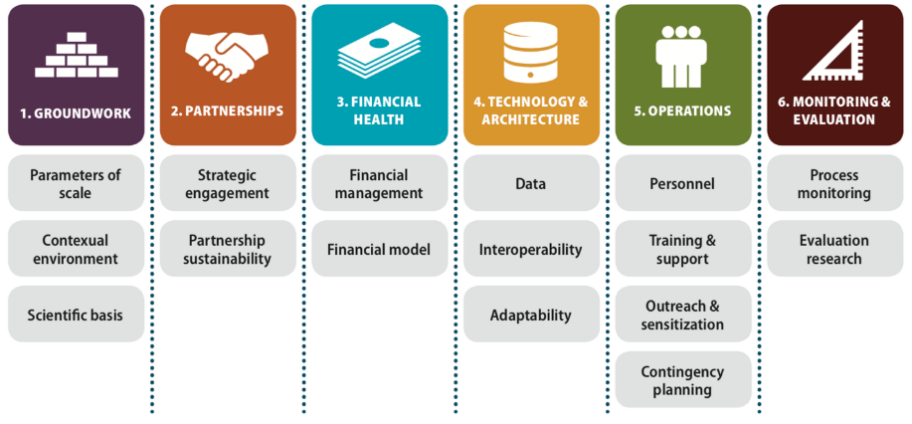
The mHealth Assessment and Planning for Scale Toolkit drivers of success within each axis of scale [13].

The MAPS Toolkit served as the conceptual framework for this study to ground Vietnam’s experience in scaling up an EIR within known factors that influence successful scale-up and sustainability of digital health products. Although the toolkit was designed to prospectively guide iterative program implementation, it has also been used for retrospective program evaluation [24]. MAPS defines the key determinants of success, also known as the “axes of scale,” to be groundwork, partnerships, financial health, technology and architecture, operations, and monitoring and evaluation (M&E) (Figure 1), each of which are divided into more specific drivers of success (Figure 2) [13].

### Setting

**Figure 3:**
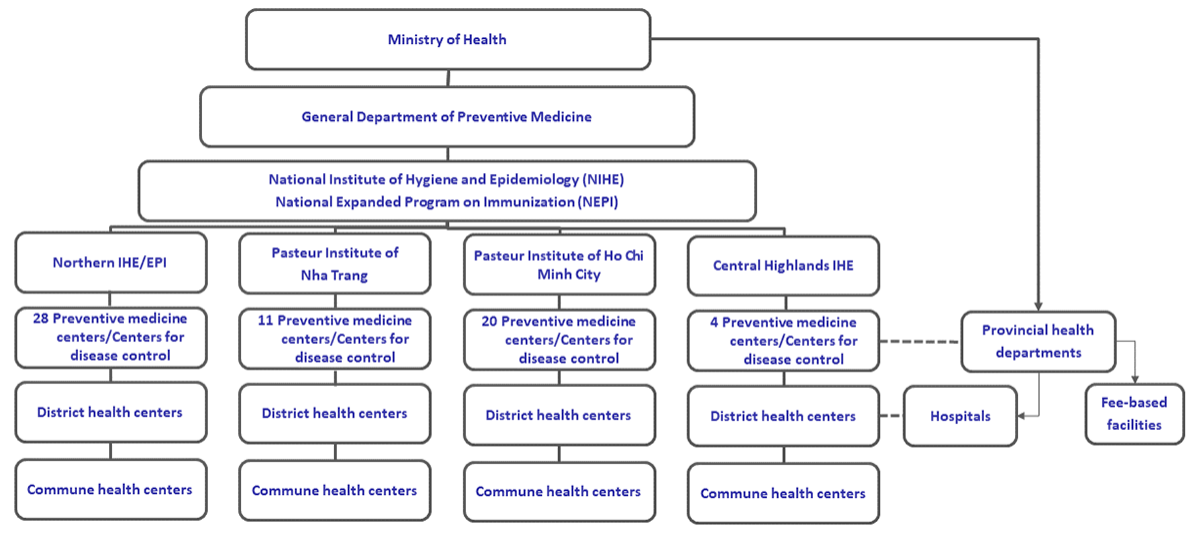
Structure of the health system in Vietnam. EPI: Expanded Program on Immunization; IHE: Institute of Hygiene and Epidemiology.

Vietnam has a 5-level health system, with administrative levels at the national, regional, provincial, district, and commune levels [25]. At the national level, the Ministry of Health (MOH) sets national policies and programs and manages the national and regional-level hospitals and institutes [26]. The provincial level oversees provincial health departments and provincial health centers and hospitals that follow national MOH policies [26]. The Vietnam National Expanded Program on Immunization (NEPI) was first introduced in Vietnam in 1981 with the primary goal of protecting children from the most common infectious diseases by providing free immunization services to children [27]. At the national level, the MOH’s General Department of Preventive Medicine (GDPM) oversees NEPI activities in 4 regions, 63 provinces and cities, 696 districts, and 11,138 communes (Figure 3) [25]. Public immunization services are mainly delivered at the commune level, the hepatitis B vaccine is provided at hospitals, and vaccines that are not supported by NEPI are administered at fee-based immunization facilities [25]. Until 2009, Vietnam’s immunization records and vaccine supply tracking were paper based. Health centers began to be equipped with computers in 2005; internet connection at the commune level became available in 2012.

### Intervention

**Figure 4:**
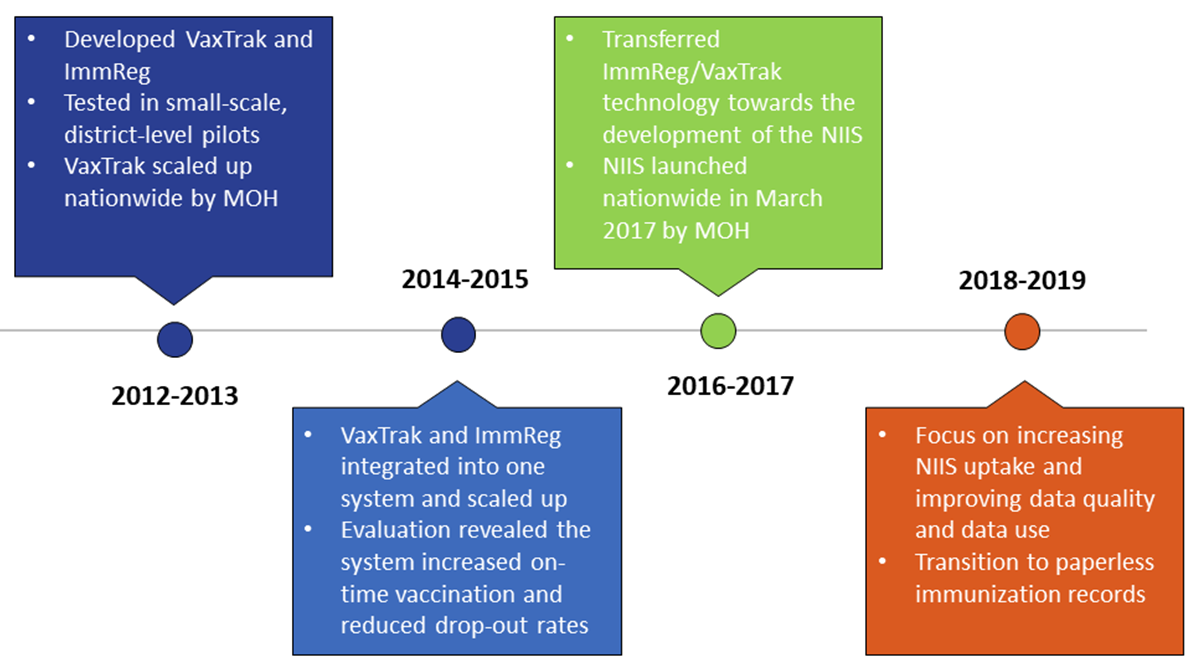
Timeline of the electronic immunization registry introduction and scale-up in Vietnam. MOH: Ministry of Health; NIIS: National Immunization Information System.

The evolution of the current national EIR in Vietnam began with a small pilot (Figure 4). From 2010 to 2012, NEPI and PATH, an international nongovernmental organization serving as a technical partner, collaborated with the WHO to develop and pilot an electronic vaccine stock management system (VaxTrak) in 3 provinces and an immunization registry software (ImmReg) in one district of Ben Tre province. The goal of the software was to improve the ability to track babies who were due for vaccination and reduce the time for immunization recording and reporting compared with a paper-based system. Both systems were successful in reducing the time burden of reporting among health workers and most users found the systems to be acceptable and feasible for scale-up [7].

These two systems were combined, upgraded, and then deployed by NEPI with support from PATH in all districts of Ben Tre province from 2014 to 2015. This resulted in increased full immunization coverage and improved on-time vaccination rates [12].

In 2016, this combined system was integrated into the NIIS, which was being developed by the MOH in partnership with Viettel, the largest mobile network operator (MNO) in Vietnam. The NIIS was to be an EIR system in which health workers could register and track the immunization records of pregnant women and newborns, as well as inform vaccine stock management. The NIIS technical working group includes GDPM, NEPI, Viettel, and PATH, who partnered to pilot the NIIS in 5 provinces. The NIIS was further upgraded based on learnings from this experience and in preparation for national scale-up. In June 2017, the NIIS was officially deployed nationally. Leading up to nationwide scale-up, the MOH requested all communes to back enter the full vaccination history for all children born from January 2015 through June 2017.

From 2018 to 2019, the NIIS technical working group focused on increasing NIIS uptake, improving data quality and data use, and transitioning completely to paperless immunization records. As of January 2020, over 20 million records have been registered into the system (including back-entered data, immunization data for pregnant women, and childhood immunizations delivered among the annual birth cohort of approximately 1.7 million [28]).

### Participants

Project documents were selected for inclusion in the document review based on project and study relevance. A total of 1 project proposal and 8 project evaluation reports detailing the project objectives, processes, successes, and challenges were included in the document review. Documents included end users’ perspectives on the acceptability and feasibility of the system through surveys of Expanded Program on Immunization (EPI) staff at provincial, district, and commune health centers conducted in 2012 and 2015.

Critical case purposive sampling [29] was used to select key informants to interview. A case was identified as critical based on their essential and extensive involvement with the project, with consideration for diversity of perspectives based on each participant’s role on the project. A total of 6 key informants were selected: 2 Ministry of Health staff (1 from GDPM and 1 from NEPI), 2 representatives from Viettel, and 2 PATH staff members. These key informants represented each of the organizations in the national-level NIIS technical working group (TWG), had been involved in every phase of the NIIS implementation, and were selected for their ability to speak to all the axes of scale in the MAPS framework.

### Data Collection

PATH staff conducted 6 in-depth semistructured interviews in person with key informants from October to December 2019. The semistructured interview guide was developed based on the evaluation questions and the MAPS framework. Interviews were audiorecorded with permission from the informant, and the interviewer documented summary notes during the interview. Audiorecordings were transcribed into Microsoft Word and translated into English.

### Data Analysis

An a priori codebook was developed, guided by the MAPS axes. The codebook was then refined by coding data from project documents and in-depth interviews in Microsoft Excel and Atlas.ti (version 8; ATLAS.ti Scientific Software Development GmbH). Coding was completed independently and concurrently by 2 PATH staff and an external evaluator. Codes were reviewed by the evaluation team and any changes or disagreements were discussed until resolved. Once coding was finalized, codes were grouped into key themes guided by the evaluation questions and MAPS axes. Exemplary quotations were identified to present the themes and were validated with key stakeholders.

### Ethics

The study procedures were reviewed and received nonresearch determination by PATH. Prior to the interviews, participants were informed of the study’s objectives, advantages and disadvantages of participating, and rights of participants. Importance of maintaining confidentiality was emphasized during training of data collectors and the start of interviews. Written consent was obtained from all participants. Study data were stored in an access-restricted server, only available to study staff for the purpose of data analysis.

## Results

### Overview

Table 1 summarizes the key facilitators and barriers experienced during scale-up of the NIIS, organized by the MAPS axes of scale.

**Table 1:** Key facilitators and barriers to scale-up.

Axes of scale	Facilitators	Barriers
Groundwork and monitoring and evaluation	- Learnings from multiple pilots and phases contributed to optimization of all other axes - Learnings established scientific basis for scale-up	N/A^a^
Partnerships	- Each member of the partnership plays a critical role - MOH^b^ is owner and leader - MOH contracts MNO^c^ to provide a service - Expansive technological, network, human, and financial resource capacity of MNO - MNO well established and trusted by government and public	N/A
Financial health	- Estimated time and cost of system - MNO provides services free of charge - Government commitment to allocate budget	- Unanticipated operational costs, particularly for refresher trainings, supportive supervision, and regular system upgrades
Technology and architecture	- User-friendly system - System adaptability - Strong data security and privacy - Existing infrastructure	- Enormity of data affects system capacity and network connectivity - Lack of national ID
Operations	- Availability of pretested and enhanced training materials and standard operating procedures - Cascading training-of-trainer training for nationwide training and technical support network - Integration of supervision within existing structures	- Dual reporting systems (paper and digital) - Low computer literacy of end users - High turnover of health care workers

^a^N/A: not applicable.

^b^MOH: Ministry of Health.

^c^MNO: mobile network operator.

### Key Facilitators to Scale-up

The partnership and operations axes were most commonly perceived to be critical to the successful deployment and scale-up of the EIR in Vietnam. Though key informants identified elements in the financial health axis and technology and architecture axis that were facilitators to successful scale-up, these axes were not as commonly or strongly mentioned in comparison to the partnership and operations axes. The groundwork and monitoring and evaluation axes were considered to be strong contributors in the success of all the other axes.

#### Partnership

The NIIS is a product of a hybrid model, with the public, private, and civil society sectors contributing to the successful scale-up and sustainability of the NIIS. The NIIS partnership has enabled the government to own and run the system alongside supportive partners working together as a team.

The NIIS TWG was formed in 2016 with the aim to contribute to the technical implementation and sustainability of the NIIS. Membership comprises MOH’s GDPM as the management authority, NEPI as the immunization expert, Viettel as the technology expert, and PATH as the liaison, serving as the connection across stakeholders. Initially the TWG met weekly, then over time it shifted to once or twice per month, depending on whether NIIS operationalization challenges emerged that required a timely response.

The most commonly expressed theme among key informants was the importance of the partnership in the success of the EIR implementation and scale-up. Key informants repeatedly noted the importance of the role that each member held in the TWG. One informant commented:

Each member of the TWG plays an important role. It could not be as it is without each member.

Key Informant A

Key informants described the importance of the MOH’s role, both to sustainably lead and to oversee the national system. As key informants observed:

Political commitment is key. First you need to have political commitment, to invest in resources and provide guidance from the government for implementation.

Key Informant B

Only the MOH can manage such a large system that includes so much of the population.

Key Informant C

NEPI provided immunization expertise and acted as the technical lead to define and develop immunization workflows, user requirements, and reporting systems and mechanisms. NEPI also oversaw and strengthened the implementation of the NIIS.

Given the expansive technological requirements of a national EIR, the MOH sought to partner with an MNO that already had an extensive network and was capable of high-capacity data storage. In addition to these technical specifications, key informants noted the importance of selecting a well-established MNO with a history of success:

If you want to implement a system at this scale, it is imperative to work with a big company like Viettel. The MNO of choice must have large capacity and a large presence in the country, be financially secure, and be able to provide necessary human resources in both quality and quantity. Choosing an MNO, it would be best to select the largest MNO with the longest history of success in the country of implementation.

Key Informant D

Additionally, some informants observed that the nature of the partnership between MOH and Viettel was a driver for successful scale-up and sustainability. That is, Viettel provided key development, storage, and maintenance services rather than only providing software developer services that would eventually be transitioned to the government to maintain and upgrade. As one informant explained:

Viettel provides a service, not just the software, so there is always a partnership and not just a handover. So, whenever the system needs to be updated, the MOH and Viettel are working together. NIIS belongs to the MOH and Viettel provides a service. This is a good model for other countries: outsource the service to a mobile network operator.

Key Informant E

PATH has played a critical role throughout the pilot–to–national scale-up process. Having both local and global experience in piloting, scaling up, and evaluating digital health products, PATH was able to transfer all technology, such as data flow and database structure design, to Viettel and share lessons learned with the TWG. PATH could also act as a liaison between the MOH, a health system–focused entity, and Viettel, an information technology entity, given its experience in global health and digital technologies. One informant explained:

PATH complements and supplements the gaps in MOH and Viettel. PATH’s experience in the field, piloting and demonstrating and disseminating that information globally, has been critical.

Key Informant B

This partnership, in which each partner contributed a critical role, was built on a foundation of trust, which key informants identified as an important driver of successful scale-up and sustainability:

Viettel is government owned, so they trust the government, and the government trusts them. So when MOH trusts PATH, then there is a big circle of trust… An MNO that is trusted by the government is very important. Because the health information is very sensitive. So the partnership can only be established with an MNO that is an old and trusted one, not a new or just established MNO. This helps with scaling-up.

Key Informant B

#### Operations

Another recurrent theme across the interviews was the strength of the end user training and support approach that was developed for successful scalability and sustainability.

For NIIS national deployment, training materials and standard operating procedures, which had been developed, tested, and enhanced based on learnings from a landscape assessment and monitoring and evaluation of multiple phases of the EIR, were readily available for use. Additionally, the cascade approach to conduct training-of-trainer trainings had been developed to quickly establish and expand a network to train end users across the country. As one informant explained:

You want to conduct a cascade training because you don’t have the resources to go everywhere. Instead, conduct the training-of-trainer training to provincial and district health workers, then they can provide training and technical support to end-users in hospitals, fee-based facilities, and commune health centers. This is the best method due to limited resources and the trainers will be the supervisors, so this informs them about who is the strongest and who is weak so they can provide feedback to them to improve.

Key Informant E

Through these trainings, a multilevel technical support network was established across the country. The trained provincial and district health staff acted as mentors who served as focal points for technical support at each health level and provided direct support to end users. Supportive supervision was integrated into existing structures of immunization supervision. In addition, Viettel trained district-level staff on the NIIS, who are now able to provide technical support to end users. This approach is working to sustain country ownership and sustainability of the NIIS in Vietnam.

#### Financial Health

Estimating the cost of the system and its implementation to appropriately allocate resources to scale up was critical to the success of the NIIS. After the first pilot from 2010 to 2012, a business model framework was developed to provide an overview of the proposed key partners, resources, and activities for sustaining and scaling an EIR in Vietnam. A costing model was also created to determine the financial resources needed to implement the system across multiple provinces in Vietnam.

Identified costs included software development and maintenance, training, supportive supervision and monitoring, and the cost for technical meetings after the first months of implementation. In the case of the NIIS, the MOH has not incurred the cost of software development and maintenance, as the MNO has been providing these services in kind, currently with the expectation that the pricing model may shift in the long-term.

In addition to estimating costs of the system, as a necessary component to successful EIR scale-up, it was essential to have the government commitment to allocate a budget to this work. As one informant explained:

The government needs to commit and support the implementation and [provide] direction and leadership to the local government to allocate the budget for implementation and maintenance.

Key Informant E

#### Technology and Architecture

Key informants noted 3 main technology and architecture facilitators to successful scale-up of the EIR related to the technology itself: its user-centered design, adaptability, and strong data security and privacy. Initial development of the EIR and subsequent upgrades prioritized end user needs. As such,

The system is designed with high-rate of acceptability and is user-friendly. It meets the basic requirements of end-users and it operates smoothly. These are factors to excite provinces to implement and use the system.

Key Informant F

The NIIS supports facility health workers to document and track immunization records and can easily print electronic records for storage in compliance with current regulations. Individual immunization records can be updated with vaccine doses delivered in different facilities, which is particularly important in large cities where many children receive vaccines from public and private facilities and there is a large migrant population moving between facilities.

To maintain system acceptability by ensuring smooth operation of the system, the NIIS has a mechanism to monitor system operations so that issues that appear can quickly be resolved. The system is updated regularly, and adaptations can be made to fit evolving needs. Relatedly, the system has strong data security and privacy measures to maintain public trust of the NIIS.

In addition to the NIIS design, the importance of an already existing technology infrastructure at health facilities cannot be overstated as a facilitator to the successful deployment and scale of the EIR. That is, because almost all health facilities in Vietnam already had computers and internet, and health care workers had access to computer systems, the NIIS could be developed with this starting point in mind. Had computers and internet not already been available at health facilities, the NIIS would likely have been designed differently or would have required additional resources at the outset for equipment and connectivity.

#### Groundwork and Monitoring and Evaluation

The steps taken in the early phases of the EIR and the M&E learnings from multiple EIR implementation phases set the groundwork for successful national scale-up, especially informing the planning, implementation, and optimization of the other axes of scale. Given this interconnected relationship between groundwork and M&E throughout the many implementation phases, we merged these two axes for our analysis.

Key groundwork activities included a landscape analysis and the development of a business model framework. The main aim of the landscape analysis was to evaluate how a digital registry might improve the ability to track children due for vaccinations and how it might shorten the time required for recording and reporting immunizations compared to a paper-based registry. This analysis provided a snapshot of the current policies, technical capacities, and health information systems already in place or under development in Vietnam. On the importance of conducting a landscape analysis, one informant emphasized:

It is a necessary step to look at two sides: the policy and technical sides. Before you scale-up, you must look at the policy environment and see what are the advantages, what is available, and what are the gaps. To understand what is financially necessary and what are some foreseen challenges.

Key Informant B

The analysis was particularly critical in informing the development of the pilot EIR system in 2010 but also continued to guide decisions about the operations and the technology and architecture of the EIR phases. One major takeaway from the analysis was the importance of understanding user needs to build a system that supports end users at all levels. The analysis also informed the development of the end user training plan, as it identified potential issues such as high turnover and low computer literacy of health care workers that could affect successful implementation and scale-up of the EIR.

Learnings gathered from both groundwork activities and M&E facilitated effective and efficient national scale-up:

We only had 8 months to work on the NIIS together [in 2016], but at that time we already had the business model and understood well about the business and the technical, so we could just transfer the knowledge and the technology to develop the NIIS. This shortened the time for NIIS development.

Key Informant E

The multiple phases of the EIR that preceded NIIS national scale-up created many opportunities for learning and optimization. In total, the Vietnam EIR system phased through 4 pilots and deployments from 2010 to 2017, starting with a pilot at the district level, then scaled and piloted at the provincial level, scaled again and piloted in multiple provinces, and then finally, scaled nationally. As one informant noted:

The piloting of the system is so important to get feedback from the end-users, before scale-up. And it is important to collect lessons learned as you go, to determine when you can and how you can scale-up.

Key Informant E

This continuous test-learn-optimize and scale-learn-optimize improvement cycle allowed for improvements in the operations and technology and architecture of the EIR, such that by the time it was scaled nationally, many things had been pretested and improved.

Furthermore, evaluation activities from these phases contributed to the growing evidence of the benefits of an EIR, as well as local validation that such a system was both feasible and effective in the Vietnam context. It was these quantifiable successes that catalyzed the support for nationwide scale-up. As one key informant recommended:

The pilot should be conducted in two years, to get the feedback and upgrade the system, and have enough time to evaluate the system. Two years is enough to evaluate the impact of the system rather than just evaluate the acceptability and feasibility of the system. In the short-term, end-users just want to say the good things.

Key Informant E

### Key Barriers to Scale-up

The financial health, technology and architecture, and operations axes were most commonly perceived to be barriers to the successful deployment and scale-up of the EIR in Vietnam.

#### Financial Health

Although the cost of EIR deployment was estimated during various EIR phases, there were some costs to national scale-up that were underestimated or not estimated. The majority of the initial financial model was based on costs associated with software development and maintenance. Budgets for operational costs of training, supportive supervision, and monitoring and evaluation were sometimes insufficient. For example, there was insufficient funding and human resources for EIR’s supportive supervision visits:

A low budget for supportive supervision visits made it difficult for higher level supervisors to conduct timely supervision of end-users.

Key Informant F

Local governments do not allocate budget for research and some don’t have supervision budgets, so they had to integrate [NIIS supervision] with supervision of other health areas.

Key Informant E

Another unanticipated cost to scale-up was that, because of the enormity of the data, the server has needed to be frequently updated and expanded. Through this experience, the TWG has identified the importance of accurately estimating the operational aspects of scaling up an EIR.

#### Technology and Architecture

Two key barriers to successful scale-up and sustainability were identified within the technology and architecture axis, namely issues with data transmission and storage and interoperability.

Although a sizing and infrastructure assessment was conducted to inform the server capacity and connectivity bandwidth, the substantial increase in the demand for data storage and transmission for national scale-up were not appropriately anticipated. There are nearly 20 million clients in the NIIS, and this number will continue to increase as the population grows. Informants explained:

We did not foresee just how large the data would be, how large of a number of people would be registered into the system, and how to store this data without slowing down the system. The number of people registered is growing rapidly… During times of high influx, the NIIS system is overloaded. The server needs to be updated and expanded frequently to accommodate the enormity of the data.

Key Informant F

We did not estimate the increase of data, therefore could not optimize the algorithm as well as the database infrastructure from the beginning.

Key Informant A

This, along with a large number of people using the system, has meant that the system can sometimes run slowly. The TWG is working to find a solution, which includes upgrading the server, separating the data storage and data query servers, and developing standard operating procedures in cases where the system is down. The MNO has also been working to address this issue by identifying and indexing frequently queried data files to optimize the speed of queries.

Another barrier to successful scale-up has been the lack of a national ID to develop an integrated, interoperable system. Viettel is currently working to develop a health information system that integrates all data from existing systems, including immunization, infectious diseases, and noncommunicable diseases, all of which have their own software without a common unique identifier for each client. This has been an issue even within the NIIS, with duplication of individual records. (Based on a January 2019 GDPM report, 1.2% of NIIS records were duplicates [30].) Similarly, facilities that use their own systems, such as hospitals and private facilities, are not interoperable with the NIIS. The NIIS includes barcode technology, and it has been suggested that each child’s unique barcode could serve as a national ID; however, with limited resources, there are few public facilities equipped with barcode readers and printers. Through this experience, the TWG has identified that having a national unique ID would be a facilitator to building an interoperable system.

#### Operations

There were 3 key barriers to successful scale-up and sustainability identified within the operations axis: dual reporting, low computer literacy of end users, and high turnover of health care workers.

From the beginning, there have been persistent concerns among health sector staff about whether an EIR would increase the workload of health care workers. Evaluations of the early phases of the system found that it successfully reduced the time burden of reporting among health workers; however, the current dual-reporting scenario is burdening the health workforce workload. Because a completely digital system is not mandated by the government, end users are tasked with using both the paper-based system and the NIIS to ensure data accuracy in both systems. During planning for national scale-up, a clear transition plan from paper to digital would reduce the workload for end users.

During the landscape analysis conducted in 2010, low computer literacy of end users was identified as a possible barrier to effective implementation and scale-up of an EIR. Some health care workers were not well versed in computer usage, including basic computer functions, such as typing and Microsoft Excel. Low computer literacy also meant that staff would not know how to use the data to calculate indicators and interpret results for decision making. As one key informant described:

One of the difficulties in the pilot phase was the computer skills of the health care workers. Some needed a lot of support, even in typing. We trained on everything. This was a big challenge for implementing the system.

Key Informant E

Given that the issue was identified during the landscape analysis, the project team was able to use various strategies in the development of the EIR and training approach to increase the usability of the EIR for end users:

Due to different levels of digital literacy of commune health workers, each commune must assign two people to take part in the training sessions. After the training, this peer support is very important to ensure success in implementation.

Key Informant E

A lot has changed since these early years of EIR implementation, with an increasingly computer-literate health workforce and increased computer and internet infrastructure at the commune level. This change has helped increase the accessibility and usability of the NIIS.

Another barrier to scale-up and sustainability identified during the landscape analysis was the frequent staff rotation and high staff turnover rate. Again, because this was identified during the landscape analysis, it was possible to address this during the development of the training approach. Multiple staff members at every level of the health system, including the health facility, district, provincial, and national levels, are trained. The cascading training-of-trainers approach (described in “Operations” under the “Key Facilitators to Scale-up” section) also ensures a sustainable peer-training system is in place to train new staff. Vietnam is also exploring e-learning options to train new staff.

## Discussion

### Principal Findings

While there were facilitators across all axes within the MAPS framework, the partnership and operations axes were most commonly perceived to be critical to the successful scale-up of the EIR in Vietnam. The EIR scale-up has been facilitated by a partnership comprised of public, private, and civil society actors working together toward shared goals and leveraging each partner’s expertise. It was operationalized through a cascading training-of-trainer approach, which was integrated within existing supervision structures and used training materials that were iteratively refined during each phase of implementation.

The most commonly perceived barriers to the scale-up of the EIR in Vietnam were related to the financial health, technology and architecture, and operations axes. Related to financial health, there were challenges in sufficiently estimating operational costs associated with training, supportive supervision, and monitoring and evaluation. Technology and architecture barriers included issues with the unanticipated volume of data storage and transmission demands and the lack of a national ID to support interoperability with other health information systems. Key operational barriers were mainly among end users and included challenges of dual-reporting systems, low computer literacy, and high staff turnover.

Overall, the phased approach of multiple small pilots allowed for iterative assessment and planning that strengthened all the axes and contributed to the successful scale-up.

### Implications for Scale-up and Future Research

Though the MAPS Toolkit was not designed as an evaluation framework, overall, the axes of scale and their specified definitions helped ground the analysis for this evaluation. The MAPS conceptual model acknowledges that scaling up is an “iterative cyclical process of thorough assessment, careful planning and targeted improvements” [13]. However, there is not an axis or a cross-cutting axis to represent the dynamic, continuous learning-and-improvement process that was so critical to the success of the full national-scale deployment of the NIIS. In Vietnam, each of the 4 phases of the EIR deployment from 2010 to 2017 allowed for opportunities to learn and make improvements that strengthened multiple axes. The technology itself improved with feedback from end users to improve its acceptability and functionality, operations benefitted by fine-tuning training materials and standard operating procedures, costing models were refined at various points, and with time and experience, trust between the partners continued to grow. Furthermore, the evaluation activities from each phase built the evidence base, which strengthened partners’ support to continue to scale the system. Thus, the MAPS framework was adapted during analysis by merging the M&E and groundwork axes as a way to acknowledge this interconnectedness of the two axes. When applying the MAPS framework, implementors and evaluators should strive to understand the interconnectness between and dynamic influences across axes.

Using the MAPS Toolkit allowed for comparison between the experience in Vietnam with that of Tanzania and Zambia, where a recent study explored the factors influencing the introduction and adoption of EIRs in low-resource settings using the MAPS Toolkit [24]. Tanzania and Zambia took a similar approach by starting implementation in a single pilot district or province before expanding the EIR further, which was also perceived as a facilitator of scale-up, as it allowed for opportunities to learn and iterate [24]. The value of pilot testing an EIR has been documented in the United States as well [9] and is consistent with the scale-up literature for other nondigital interventions [31].

The central role of the partnership model in Vietnam cannot be overstated. There were initial challenges as partners developed shared language and understanding across their areas of expertise, which included public health and immunization, financial health, and information technology. It was important to allow time for this learning curve and to formalize roles as part of the NIIS TWG. Lessons learned from implementing subnational EIRs in the United States also highlighted the importance of a steering committee or coalition to guide EIR development and garner support [9]. Similar to Vietnam, in both Tanzania and Zambia, the MOH leadership’s support of the EIR was an important facilitator, and the MOH relied on PATH as a technical partner. However, the partnership model in these African countries differed from Vietnam’s in that nongovernmental partners, including PATH, John Snow Inc, and the Catholic Medical Mission Board were more directly involved with implementing the EIR at subnational levels in Tanzania and Zambia [24]. Other countries may also not be able to replicate the MNO partnership that was a key facilitator to scale-up in Vietnam, as Viettel is uniquely positioned as a state-owned MNO with large market share across the country.

In terms of operations, Tanzania initially used an on-the-job training approach that relied on PATH staff to visit each health facility, but later Tanzania and Zambia implemented a training-of-trainers approach similar to Vietnam’s model. As mentioned, nongovernmental partners were more heavily involved in the initial subnational deployment and trainings in Tanzania and Zambia, which was also a large cost driver in these countries [32]. In all three countries, EIR supervision was integrated within existing structures, which was a facilitator for scale and sustainability, but also meant there was limited time spent specifically on EIR issues during supervision visits. Similar to Vietnam, Tanzania and Zambia also faced barriers due to dual-reporting systems, low computer literacy of end users, and high health care worker turnover. Other studies echo that human resources are a challenge to the Vietnam EPI as a result of limited resources for supervision in hard-to-reach areas, underpaid and unmotivated frontline workers, and low levels of knowledge and computer literacy [33].

Whereas in Vietnam, the existing technology infrastructure was in place at health facilities, this was not the case in Tanzania and Zambia, where lack of hardware and electricity was a barrier to initial introduction of the EIR [24]. Other technology and architecture barriers to scale of the EIRs in Tanzania and Zambia included synchronization delays, discrepancies in data across systems, and challenges due to separate immunization service delivery and stock management systems [24]. In Vietnam, this last challenge was avoided by integrating ImmReg and VaxTrak into a single system (NIIS) before scaling nationwide. The iterative piloting of the technology in Vietnam may have avoided the heavy costs that can be associated with EIR redesign or reconfiguration [9,32]. Both Tanzania and Zambia had to modify their EIR requirements and design a second system for scale [34].

As more countries aim to introduce EIRs, these findings can inform efforts to plan for scale-up. Seth Berkley, CEO of Gavi, the Vaccine Alliance, wrote about the importance of harnessing digital innovations to support immunization, noting that “one of the biggest needs is for affordable, secure digital identification systems that can store a child’s medical history, and that can be accessed even in places without reliable electricity” [35]. Previous studies have recommended using digital health innovations for immunization, based on systematic reviews [36], but there is recognition that current research on the role of digital health in immunization initiatives is limited [22,36]. This is true for the field of digital health overall, with repeated calls to continue to strengthen the evidence base [6]. The findings presented here touch on priority evidence gaps related to evaluating economics, enabling ecosystems, financial and programmatic sustainability, and data use pathways related to digital health interventions [6].

### Limitations

This study was based on the experience scaling the EIR in Vietnam, and care should be taken when generalizing these findings beyond Vietnam. This limitation was in part addressed by identifying determinants guided by the MAPS Toolkit, a conceptual framework that can be applied to digital health implementations in varied contexts.

This study purposively selected key informants to represent the range of partners on the NIIS TWG; each was very involved in the EIR scale-up and could speak to the details of the implementation. The study focused on national-level stakeholders and did not include key informants representing subnational or community perspectives. Although end user perspectives were represented in the documents reviewed, for a more comprehensive understanding of the factors influencing the EIR scale-up in Vietnam, future studies could also include perceptions of the barriers and facilitators from EIR end users at the district and health facility levels, as well as caregiver or community perceptions of immunization service delivery. Different determinants may emerge at each level. For example, a systemic literature review (with the majority of studies from the United States) identified health care providers’ perceptions of usefulness and ease of use of digital health innovations as influential factors to their acceptance of the innovation [37]. Dolan et al [24] highlighted some of the barriers influencing EIR scale-up at subnational levels in Tanzania and Zambia, including inadequate data bundles, increased workload due to dual systems, and lack of EIR integration with the health management information system (Zambia only). In Vietnam, subnational differences in urbanization, socioeconomic status, behaviors, and other factors that may have affected EIR scale-up were not the focus of this study.

### Conclusion

This study described the key facilitators and barriers that influenced the scale-up of the NIIS in Vietnam using a comprehensive digital health framework. The results highlight the importance of the measured, iterative approach that was taken to gradually expand a series of small pilots to nationwide scale. Key facilitators included the partnership model, which leveraged complementary strengths of the MOH and GDPM, NEPI, Viettel, and PATH, and the operational approach to introducing the NIIS with lean, iterative, and integrated training and supervision. Key barriers to scale-up included insufficient estimates of operational costs, unanticipated volume of data storage and transmission, lack of a national ID to support interoperability, and operational challenges among end users. The findings from this study can be used to inform other countries considering, introducing, or in the process of scaling an EIR or other digital health innovations.

## Abbreviations

BMGF: Bill and Melinda Gates Foundation
EIR: electronic immunization registry
GDPM: General Department of Preventive Medicine
MAPS: mHealth Assessment and Planning for Scale
M&E: monitoring and evaluation
MNO: mobile network operator
MOH: Ministry of Health
NEPI: The Vietnam National Expanded Program on Immunization
NIIS: Vietnam’s National Immunization Information System
TWG: technical working group
WHO: World Health Organization
